# Cryo-EM structure of arabinosyltransferase EmbB from *Mycobacterium smegmatis*

**DOI:** 10.1038/s41467-020-17202-8

**Published:** 2020-07-07

**Authors:** Yong Zi Tan, José Rodrigues, James E. Keener, Ruixiang Blake Zheng, Richard Brunton, Brian Kloss, Sabrina I. Giacometti, Ana L. Rosário, Lei Zhang, Michael Niederweis, Oliver B. Clarke, Todd L. Lowary, Michael T. Marty, Margarida Archer, Clinton S. Potter, Bridget Carragher, Filippo Mancia

**Affiliations:** 1 Department of Physiology and Cellular Biophysics, Columbia University, New York, NY 10032 USA; 2 National Resource for Automated Molecular Microscopy, Simons Electron Microscopy Center, New York Structural Biology Center, New York, NY 10027 USA; 3 Instituto de Tecnologia Química e Biológica António Xavier, Universidade Nova de Lisboa (ITQB NOVA), 2780-157 Oeiras, Portugal; 4 Department of Chemistry and Biochemistry, University of Arizona, Tucson, AZ 85721 USA; 5 Department of Chemistry, University of Alberta, Edmonton, Alberta T6G 2G2 Canada; 6 Center on Membrane Protein Production and Analysis, New York Structural Biology Center, New York, NY 10027 USA; 7 Department of Microbiology, University of Alabama at Birmingham, Birmingham, AL 35294 USA; 8 Department of Anesthesiology, Columbia University, New York, NY 10032 USA; 9 Institute of Biological Chemistry, Academia Sinica, Academia Road, Section 2, #128, Nangang, Taipei, 11529 Taiwan; 10 Bio5 Institute, University of Arizona, Tucson, AZ 85721 USA; 11 Simons Electron Microscopy Center, New York Structural Biology Center, New York, NY 10027 USA; 12 Department of Biochemistry and Molecular Biophysics, Columbia University, New York, NY 10032 USA

**Keywords:** Transferases, Tuberculosis, Cryoelectron microscopy

## Abstract

Arabinosyltransferase B (EmbB) belongs to a family of membrane-bound glycosyltransferases that build the lipidated polysaccharides of the mycobacterial cell envelope, and are targets of anti-tuberculosis drug ethambutol. We present the 3.3 Å resolution single-particle cryo-electron microscopy structure of *Mycobacterium smegmatis* EmbB, providing insights on substrate binding and reaction mechanism. Mutations that confer ethambutol resistance map mostly around the putative active site, suggesting this to be the location of drug binding.

## Abstract (web summary)

The cryo-EM structure of *Mycobacterium smegmatis* arabinosyltransferase B EmbB involved in mycobacterial cell wall biosynthesis provides insights into the substrate binding and reaction mechanism. Mapping of the ethambutol resistance associated mutations onto the structure suggests the location of the drug binding site.

## Introduction

**Fig. 1:**
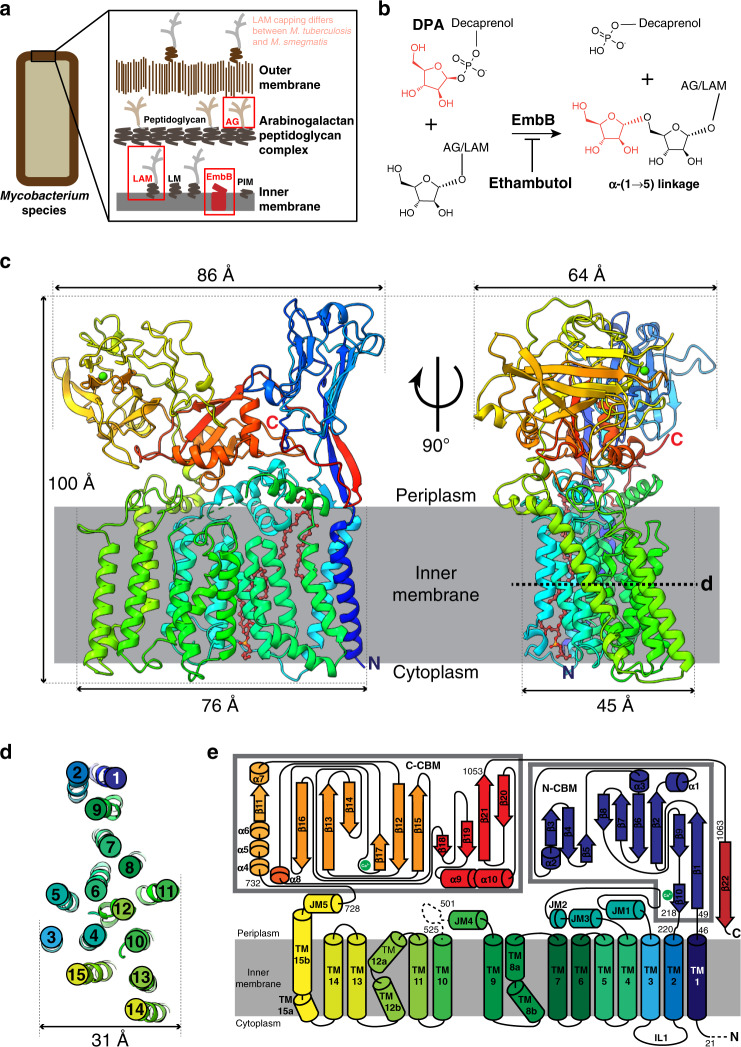
Cryo-EM structure of EmbB. **a** Model of the cell envelope of mycobacterial cell envelope based on Dulberger et al.^75^. Red boxes highlight arabinogalactan (AG) and lipoarabinomannan (LAM) components, synthesized by EmbA/EmbB (red) and EmbC, respectively. LAM for *M. tuberculosis* is capped by α-mannose glycans while in *M. smegmatis* it is capped by phosphoinositol instead^76^. **b** Reaction catalyzed by EmbB, which is inhibited by ethambutol. **c** Single-particle cryo-EM structure of EmbB, rendered in cartoon form and colored in rainbow from N terminus (blue) to C terminus (red). A Ca^2+^ ion is shown as a green sphere. The bound lipids are represented as ball-and-sticks, colored in brown. Two orthogonal views that are perpendicular to the plane of the membrane are shown. Membrane boundaries were derived from the interface between the nanodisc lipids and solvent. **d** Arrangement of the TM helices of EmbB, viewed as a slice in the plane of the membrane, as indicated in (**b**) and magnified. **e** Two-dimensional topological diagram of EmbB. The two CBMs are enclosed in separate gray boxes. The topology diagram is rainbow colored from N terminus (blue) to C terminus (red). Unbuilt parts of the model, due to poor map density, are indicated by dotted lines. Bound Ca^2+^ atoms are shown as green circles.

The cell envelope is crucial for growth and virulence of pathogenic mycobacteria like *M. tuberculosis*^1^ and is a major contributor to resistance against common antibiotics^2^. Its main component is the mycolyl-arabinogalactan-peptidoglycan complex, which consists of peptidoglycan, a branched heteropolysaccharide arabinogalactan (AG) and long chain mycolic acids (Fig. 1a). Another major component is the lipidated heteropolysaccharide lipoarabinomannan (LAM)^3^. Of the enzymes involved in mycobacterial cell wall biosynthesis, arabinofuranosyltransferases are responsible for the addition of D-arabinofuranose sugar moieties to AG and LAM^2^. These transmembrane (TM) enzymes utilize decaprenylphosphoryl-D-arabinofuranose (DPA) to transfer an arabinofuranose unit to the growing lipidated polysaccharides of the cell envelope^4^.

Arabinosyltransferase B (EmbB), a 117 kDa integral membrane enzyme involved in the α-(1→5)-linked extension of the AG arabinan chain (Fig. 1b), is one of the best characterized members of the aforementioned family^5,6^. Its gene belongs to an operon coding for two other homologous arabinosyltransferases EmbA (40% identity between *M. smegmatis* EmbB and EmbA, 42% identity in *M. tuberculosis*, acts also on AG) and EmbC (46% identity between *M. smegmatis* EmbB and EmbC, 44% identical in *M. tuberculosis*, acts on LAM) (Supplementary Figs. 1 and 2). The operon was named due to the sensitivity of these gene products to ethambutol, a first-line antibiotic against tuberculosis^7^ and nontuberculous mycobacterial (NTM) disease^8^. EmbB and EmbC have mutations known to lead to ethambutol resistance^9^, while a clear effect of the drug on EmbA activity has not been established^10^. The dearth of atomic models of these proteins—only a high resolution structure of the C-terminal soluble domain of EmbC is available^11^—has thus far hindered our understanding of the catalytic action and drug resistance mechanisms of these proteins, although this situation has been recently remedied to a certain extent by eludication of structures of the EmbA–EmbB and EmbC–EmbC dimer complexes^12^. Here, we report the structure of *M. smegmatis* EmbB to 3.3-Å resolution, providing insights on substrate binding and reaction mechanism. Mutations that confer ethambutol resistance map mostly around the putative active site, suggesting this to be the location of drug binding.

## Results and discussion

### Cryo-EM structure of *M. smegmatis* EmbB

To better understand the function of EmbB at a molecular level, we adopted a structural genomics approach that identified the *M. smegmatis* ortholog (68% identical in *M. tuberculosis*), out of 14 screened, as a suitable candidate for structural studies based on expression levels in *E. coli* and stability in detergents compatible with structure determination^13^. Using single-particle cryogenic electron microscopy (cryo-EM), we determined the structure of EmbB from *M. smegmatis* expressed in *E. coli* and reconstituted in lipid-filled nanodiscs to 3.3 Å resolution (Supplementary Figs. 3–5 and 6a, and Table 1). Here, EmbB appears as a monomer, consisting of 15 TM helices and two distinct periplasmic carbohydrate binding modules (CBMs) (Fig. 1c–e). The first two TM helices are not found in other glycosyltransferase structures solved to date, and seem to serve to anchor the N-terminal CBM (N-CBM) to the membrane. The next 11 TM helices adopt a typical GT-C glycosyltransferase fold^14^, structurally similar to enzymes from various glycosyltransferase families: mycobacterial AftD from GT53^15^, archaeal ArnT from GT83^16^, yeast Pmt1-Pmt2 from GT39^17^, and bacterial PglB from GT66^18^ (Supplementary Fig. 7a–f). The last two TM helices are shared only with ArnT. Thereafter, the polypeptide chain exits the membrane in the periplasm to form the second C-terminal CBM (C-CBM). The C-CBM then loops back around to complete a β-sheet with the N-CBM, likely to secure the N-terminal domain in place.

**Table 1:** Cryo-EM data collection and modeling statistics EmbB.

	Session 1	Session 2
Microscope	FEI Titan Krios (same microscope)	
EM data collection/processing		
Magnification	120,000	37,000
Voltage (kV)	300	
Camera	Falcon III	Gatan K2 Summit
Mode	Counting	Counting
Set defocus range (μm)	0.5–2.5	0.3–2.9
Defocus mean ± std (μm)	1.8 ± 0.29	1.9 ± 0.27
Exposure time (s)	86.4	8
Number of frames	80	80
Dose rate (e−/pixel/s)	0.4	4.3
Total dose (e−/Å^2^)	78.02	77.53
Pixel size (Å)	0.665	0.667
Number of micrographs	2158	7833
Number of particles (after initial cleanup)	162,271	700,201
Number of particles (in final map)	57,970	
Symmetry	C1	
Resolution (global) (Å)	3.3	
Local resolution range	2.8–16.0	
Directional resolution range	3.0–3.4	
Sphericity of 3DFSC	0.99	
SCF value^a^	0.98	
Map sharpening b-factor (Å^2^)	−72.5	
Model statistics		
Initial model used (PDB code)	3PTY	
Map-to-model resolution (Å)	3.4	
Model composition		
Non-hydrogen atoms	15,844	
Residue range	21–500, 526–1082	
Ligands	4	
Map CC	0.743	
RMSD [bonds] (Å)	0.0065	
RMSD [angles] (Å)	1.21	
All-atom clashscore	2.67	
*B* factors (Å^2^)		
Protein	44.96	
Ligands	44.63	
Ramachandran plot		
Favored (%)	94.87	
Allowed (%)	5.05	
Outliers (%)	0.08	
Rotamer outliers	0.00	
C-β deviations	0	
MolProbity score	1.41	
EM-Ringer score	3.14	

^a^The SCF value is calculated as described^66^, but currently assumes that all orientations have been properly assigned and does not take into account false positive assignment.

### Mapping of the putative active site

**Fig. 2:**
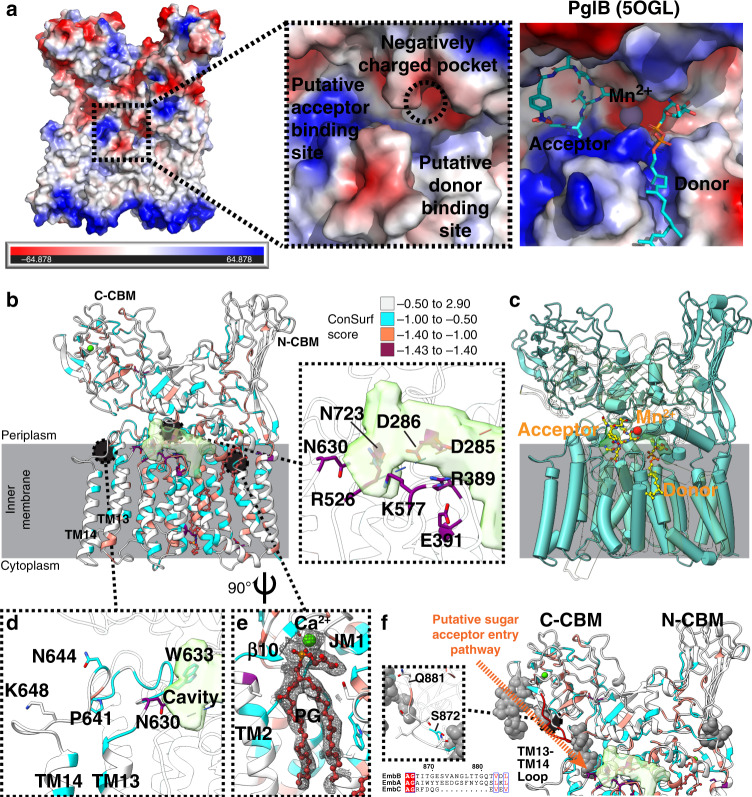
Structural features of EmbB. **a** Electrostatic representation of EmbB, with a zoom-in around the putative active site, labeled according to where the substrates are likely to bind, where red is more negatively charged and blue more positively charged. A comparison with the PglB active site with bound substrates is also shown, after PglB was aligned against EmbB. **b** Structure of EmbB, rendered in cartoon and colored based on ConSurf^69^ score for sequence conservation. The more negative the score, the more conserved the residue. The putative active site cavity, generated by the Voss Volume Voxelator server^68^ is colored in semi-transparent green. The insert shows the putative active site cavity with the strictly conserved residues labeled. **c** EmbB (pale blue) is superimposed on PglB (semi-transparent yellow), with the ligands and Mn^2+^ ion of PglB shown in yellow as sticks and a ball, respectively. **d** Residues that are known to maintain catalytic activity while altering substrate specificity on a loop between TM13 and TM14 are labeled and side chains are shown. **e** A tightly bound phosphatidylglycerol (PG shown as ball-and-stick) and calcium ion (shown as a ball) in a pocket between TM2, JM1, and β10 is shown. The density map of the lipid and the ion is displayed as mesh. **f** Glycan ligands for the top ten Dali server hits for both N-CBM and C-CBM were mapped onto the structure as a gray ball representation. 2WJS [10.2210/pdb2WJS/pdb] (PDB ID) was used for N-CBM, while 3PTY [10.2210/pdb3PTY/pdb] and 4GWM [10.2210/pdb4GWM/pdb] were used for the C-CBM. The missing loop in EmbC is colored in red, and the insert is a zoomed-in view. Sequence alignment of the region around the loop is appended below the insert. The putative sugar acceptor entry pathway is shown as an orange dotted line.

EmbB has a single large cavity (volume of ~1120 Å^3^) at the membrane–periplasm interface that encompasses juxtamembrane (JM) helix 4 and a disordered stretch of around 20 residues between JM4 and TM10 (Fig. 2b and Supplementary Fig. 6b). Conservation analyses reveal that this cavity contains highly conserved charged residues (D285, D286, R389, E391), where the residues corresponding to D285 and D286 are required for catalytic activity of corynebacterial EmbB (D297 and D298)^19^ and mycobacterial EmbC (D293 and D294)^20^ (Fig. 2a). The putative active site has a number of negatively charged amino acids—D285, D286, and E313—which would help stabilize the carbon with a partial positive charge in the anticipated exploded S_N_2-like transition state^21^. Structural alignments with the other GT-C structures all show superimposition of their active site with this cavity. For instance, in PglB, both the donor, the acceptor and a catalytic Mn^2+^ localize here (Fig. 2a, c). Based on this structural comparison, the lipidic donor (DPA) is likely to bind in the pocket formed by TM helices 7–9, on the right side of the cavity, with the soluble acceptor substrate binding to the left. As this structure was determined in the absence of any bound ligands, we expect the disordered residues between JM4 and TM10 to become ordered upon substrate binding, akin to what was shown for the PglB EL5^18^ and ArnT PL4 loops^16^ (Supplementary Fig. 7e, f).

While active site mutants in EmbB result in suppressed bacterial growth^19^, a series of mutations (N630, W633, P641, N644, K648) have been shown to retain enzymatic activity yet reduce incorporation of arabinose, resulting in the formation of a truncated AG^19^. Similar findings have been reported for LAM in EmbC^20,22^. All these residues map to the loop between TM helices 13 and 14, situated at the entrance of the cavity for the putative sugar acceptor (Fig. 2d), suggesting a role for it in regulating the oligosaccharides that can act as acceptors for EmbB^19,20^.

### Presence of tightly bound lipids in EmbB

We observe two bound lipids in our structure, in a pocket formed by TM helices 2, 5, 6, 7, and 9 on opposite leaflets (Fig. 1c). The lipid on the outer leaflet appears to have a cation bound to its head group, mediating extensive interactions with the backbone and side chains of residues from TM2, JM1, and β10 (Fig. 2e, Supplementary Fig. 7g). To identify these lipids, we performed native mass spectrometry (MS) of EmbB, which showed that EmbB appeared as a dimer when solubilized in detergent C12E8 with a series of bound molecules with masses around 300–350 Da (Supplementary Fig. 8a). In addition, there were two peaks that showed larger abundances, and we hypothesize that these corresponded to two or three bound lipid molecules with masses of ~750 Da (Supplementary Fig. 8a). To remove bound adducts, we also performed denatured liquid chromatography–MS analysis. Under denaturing conditions, EmbB retained a tightly bound calcium ion (Supplementary Fig. 8b). An additional peak was observed with 749 ± 21 Da mass, which also partially retained a calcium ion. This mass is consistent with a bound phosphatidylglycerol (PG), which is also present in mycobacterial inner membranes (average molecular weight 761.073 Da)^23,24^. These lipids (or equivalent ones endogenously) may be important in stabilizing the protein structure (Supplementary Fig. 7g).

### EmbB has two carbohydrate binding modules

In the periplasmic region, the two CBMs exhibit β-sandwich folds. Using the top ten hits from the Dali server^25^, structurally similar CBMs were aligned with the two CBMs of EmbB. This allowed us to interrogate the possible glycan-binding locations; potential substrates that were sterically hindered in the EmbB structure were discarded. As expected, the EmbB C-CBM structure is highly homologous to the corresponding CBM in EmbC (PDB ID: 3PTY [10.2210/pdb3PTY/pdb])^11^, with an RMSD of 1.2 Å. N-CBM’s structural homologs bind to at most one monosaccharide unit, whereas structural homologs of the C-CBM reveal binding to more complex glycans, corroborating what was previously observed for the EmbC C-CBM^11^. The co-crystallized Ara-(1 → 5)-Ara-O-C8 ligand for the EmbC C-CBM maps to the loop of EmbB between TM helices 13 and 14, which we earlier proposed to control access of the acceptor to the active site (Fig. 2f). This suggests a pathway for the binding of the acceptor in the active site exclusively via the C-CBM (Fig. 2f). By screening purified EmbB against a synthetic array of mycobacterial glycan fragments^26^, we found that the protein preferentially binds highly-branched arabinose-containing oligosaccharides (Supplementary Fig. 9), supporting the hypothesis that the C-CBM binds to multiple monosaccharide residues.

A comparison of EmbA, EmbB, and EmbC sequences revealed that a loop region spanning from S872 to Q881 is missing in EmbC (Fig. 2f and Supplementary Fig. 2). In EmbB, this loop is located directly above the TM13-14 loop and is part of the C-CBM; it is in the path we propose the acceptor might take to bind. This could explain the different substrate specificities reported of the Emb family members: EmbA and EmbB act specifically on AG, while LAM is a substrate of EmbC, even though all enzymes catalyze the same reaction: addition of an arabinose residue α-(1→5) to an existing arabinan chain^1^.

### Ethambutol resistance mutations map to putative active site

**Fig. 3:**
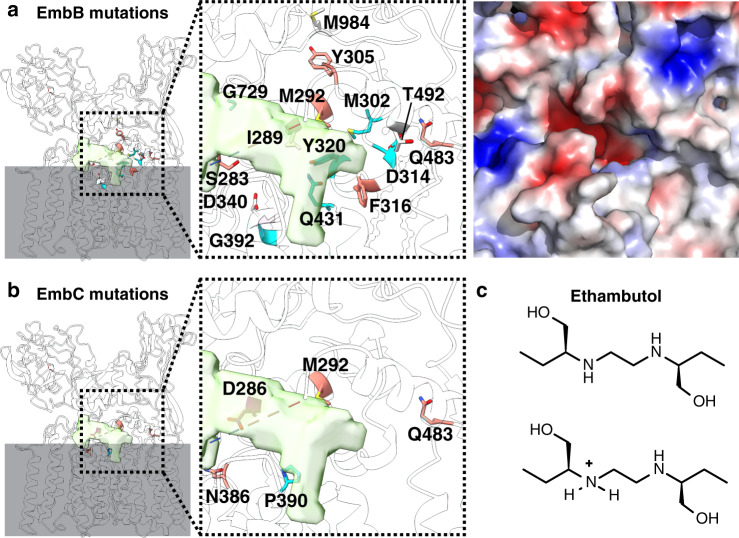
Ethambutol resistance mutations of EmbB and EmbC. Mutations known to confer resistance to ethambutol in EmbB (**a**) and EmbC (**b**) are mapped on the overall structure and as a zoom-in, with their side chains displayed and colored based on the ConSurf score as in Fig. 2. The other regions of the EmbB model are rendered semi-transparent. The electrostatic potential of the zoom-in region of EmbB is shown on the right. **c** Chemical structure of (*S*, *S*)-ethambutol, showing both its neutral and one of two single positively charged forms.

The structure of EmbB presents the unique opportunity to spatially map out data resulting from decades of known ethambutol resistance mutations in both EmbB and EmbC^9,27–30^. Focusing only on residues conserved between *M. tuberculosis* and *M. smegmatis* (Supplementary Table 1), we found that mutations causing resistance all cluster around the putative active site (Fig. 3a). Notably, these mutations are closer to the putative DPA binding site, suggesting that ethambutol might interfere with recruitment of the arabinose donor, thereby inhibiting enzyme function. Most mutated residues are not highly conserved, which both follows evolutionary logic in terms of maintaining structural integrity and function, and also provides a template to predict residues that might be susceptible to drug-induced mutations in the future. Based on the reported p*K*_a_’s for ethambutol (p*K*_a1_ = 6.35, p*K*_a2_ = 9.3)^31^, the drug is expected to be positively charged at physiological p*H*, suggesting that ionic interactions are involved in drug binding (Fig. 3c). Indeed, many of the mutations are conversions of negatively charged residues into uncharged ones (D314G/Y, D340A), or of uncharged residues into positively charged ones (Q431R, Q483K, T492R, M984R) (Fig. 3a). The only two mutations decreasing the protein overall net charge (G392D, G729D) are from residues located at the periphery of the cavity. Moreover, the homology between EmbB and EmbC enabled us to map the EmbC mutations that contributed to drug resistance onto the EmbB structure (Fig. 3b and Supplementary Table 2). Again, these mutations cluster around the putative DPA binding site. Surprisingly though, the highly conserved D286 residue is also involved in drug resistance in EmbC, which could be explained by the fact that D286G mutation reduces but does not abolish the catalytic activity of EmbC^29^.

### Comparison with heterodimeric Emb structures

Recently, the structures of heterodimeric *M. smegmatis* and *M. tuberculosis* EmbA, EmbB, and EmbC were determined^12^. *M. smegmatis* EmbB was solved as a dimer with EmbA, bound to either ethambutol or di-arabinofuranose (Supplementary Fig. 10). Compared with our monomeric, apo-structure, the disordered residues between JM4 and TM10 indeed become ordered; the rest of the structure, however, is very similar (Supplementary Fig. 10c). Whether the ordering of these residues is caused by the addition of substrates or hetero-dimerization is yet to be determined. Notably, the dimeric *M. smegmatis* EmbA–EmbB was overexpressed endogenously and surprisingly had meromycolate extension ACP (AcpM) bound, the same AcpM that is also associated to mycobacterial arabinofuranosyltransferase AftD^15^. Our monomeric EmbB structure does not have the AcpM bound likely because it was expressed heterologously in *E. coli*. AcpM in the EmbA–EmbB dimer structure extends its 4′-phosphopantetheine into the same pocket as that of inner leaflet PG present in our monomeric EmbB structure (Fig. 1c, Supplementary Fig. 10b). The lack of AcpM did not cause any significant conformational changes in the structure of EmbB around the AcpM binding site, suggesting that AcpM might not have a critical functional role, unlike what seems to occur in AftD (Supplementary Fig. 10c).

In conclusion, we report the full-length monomeric structure of a mycobacterial arabinosyltransferase from the Emb family. The structure was obtained by cryo-EM in close to the native environment by its incorporation into a lipid-filled nanodisc, and the data show that EmbB has a conserved GT-C fold. Analysis of the structure allowed us to map the putative active site as well as substrate binding sites. We localized mutations that maintain catalytic activity while altering substrate specificity to a loop between TM13 and TM14, juxtaposed to the putative active site, which we propose controls access of the acceptor to the active site. A tightly bound phosphatidylglycerol lipid and calcium cation that likely serve a structural purpose were evident in the density map, and their presence and identity were confirmed using native and denaturing mass spectrometry. Mapping of known ethambutol mutations on the structure suggests that this drug binds in close proximity of the putative active site, providing a framework to better understand if not predict resistance-causing loci. Finally, our work provides a template for future structure-based drug design efforts aimed at enhancing the efficacy of this front-line drug that is effective against tuberculosis (*M. tuberculosis*^32^, *M. bovis*^33^, *M. microti*^34^) and NTM disease^32^ (*M. avium*, *M. kansaii*^35^). This is of particular importance in the face of increasingly frequent infections with drug resistant strains of *M. tuberculosis*^7^ and other disease-causing mycobacteria^32,35–37^. Note that our solved structure of EmbB is from *M. smegmatis*, a model species for the entire mycobacteria family^37^ that is non-pathogenic. Hence, not all observations might be directly transferrable to the aforementioned pathogenic mycobacterial species, but should instead serve a guide for future studies of this family of enzymes.

## Methods

### Statistics

For calculations of Fourier shell correlations (FSC), the FSC cut-off criterion of 0.143^38^ was used. No statistical methods were used to predetermine sample size. The experiments were not randomized. The researchers were not blinded to allocation during experiments and outcome assessment.

### Sequence alignment

Protein sequences of EmbA, EmbB, and EmbC from *M. tuberculosis* and *M. smegmatis* were obtained from the Mycobrowser^39^, with the following KEGG identifiers: EmbA *Mtb*—Rv3794, EmbB *Mtb*—Rv3795, EmbC *Mtb*—Rv3793, EmbA *Msm*—MSMEG_6388, EmbB *Msm*—MSMEG_6389, and EmbC *Msm*—MSMEG_6387. The sequences were then aligned using Clustal Omega (https://www.ebi.ac.uk/Tools/msa/clustalo/)^40^ and displayed using ESPript (http://espript.ibcp.fr)^41^.

### Genomic expansion and small-scale screening

EmbB genes were identified from a collection of 14 *Mycobacterium* genomes using a bioinformatics approach^13^. Ligation independent cloning (LIC) was used to clone these targets from the genomes into five LIC-adapted expression vectors (pNYCOMPS-Nterm, pNYCOMPS-Cterm, pNYCOMPS-N23, pNYCOMPS-C23, and pMCSG7-10x) that contained a tobacco etch virus (TEV) protease cleavage site (ENLYFQSYV) and decahistidine affinity tag. Small and medium scale expression was performed in a high throughput manner as described in detail in a previous protocol by Bruni and Kloss^42^. A number of orthologs could be cloned and expressed well, but *M. smegmatis embB* was chosen over the others because it represents a model organism used to the study pathogenic *M. tuberculosis*. *M. smegmatis embB* was ultimately cloned using LIC into a pMCSG21 expression vector^43^ that contained a TEV protease cleavage site and Strep-tag on the 3′ end of the insert. This expression construct was used for all subsequent experiments.

### EmbB expression, purification, and nanodisc reconstitution

*M. smegmatis embB* in the pMCSG21 plasmid was transformed into BL21 (DE3) pLysS *E. coli* competent cells and plated onto Luria broth (LB) agar (Fisher) plates supplemented with 100 μg mL^−1^ ampicillin (Sigma) and 100 μg mL^−1^ spectinomycin (Sigma), and grown overnight at 37 °C. In the next day, a colony was picked and used to inoculate a starter culture containing 150 mL of 2xYT medium (Fisher) supplemented with 100 μg mL^−1^ ampicillin and 100 μg mL^−1^ spectinomycin. The starter culture was grown overnight at 37 °C in an incubator (New Brunswick Scientific) shaking at 240 r.p.m. The following day, six 2-L baffled flasks each with 800 mL of 2xYT medium (Fisher) supplemented with 100 μg mL^−1^ ampicillin and 100 μg mL^−1^ spectinomycin were inoculated with 10 mL of starter culture. The cultures were then grown at 37 °C shaking at 240 r.p.m. until cells reached an optical density (OD) at 600 nm of ~1.0 (~3 h). Temperature was then reduced to 22 °C and protein expression was induced by addition of 0.2 mM isopropyl β-D-1-thiogalactopyranoside (IPTG) (Fisher). The culture was then incubated overnight shaking at 240 r.p.m. The next day, the cells were harvested by centrifugation at 4000 × *g* utilizing a H6000A/HBB6 rotor (Sorvall) for 30 min at 4 °C. The supernatant was discarded and the pellet was resuspended in chilled 1x phosphate buffered saline (PBS) and centrifuged again at 4000 × *g* for 30 min at 4 °C. The supernatant was again discarded and the pellet was resuspended in lysis buffer containing 20 mM HEPES pH 7.5, 200 mM NaCl, 20 mM MgSO_4_, 10 μg mL^−1^ DNase I (Roche), 8 μg mL^−1^ RNase A (Roche), 1 mM tris(2-carboxyethyl)phosphine hydrochloride (TCEP), 1 mM PMSF, 1 tablet in 1.5 L buffer EDTA-free cOmplete protease inhibitor cocktail (Roche). For a 4.8 L of culture, the yield corresponded to ~10–20 g of wet cell pellet mass, which was resuspended with ~250 mL of lysis buffer. Cells were lysed by passing the suspension through a chilled Emulsiflex C3 homogenizer (Avestin) three times. The crude membrane fraction was isolated by ultracentrifugation at 37,000 × *g* in a Type 45 Ti Rotor (Beckman Coulter) at 4 °C for 30 min. The supernatant was discarded and the pellet was resuspended in the lysis buffer up to a volume of 240 mL and homogenized using a hand-held glass homogenizer (Konte). The membrane fraction was then stored at −80 °C until later use to purify protein.

The thawed membrane fraction was solubilized by adding n-dodecyl-β-D-maltopyranoside (DDM) to a final concentration of 1% (w/v) detergent for 2 h at 4 °C with gentle rotation. Insoluble material was removed by ultracentrifugation at 40,000 × *g* in Type 45 Ti Rotor at 4 °C for 30 min. 1.5 mg of avidin (IBA Lifesciences) was added to the supernatant to block any endogeneous biotin, and the mixture was left on ice for 5 min. Thereafter, the supernatant was added to six Falcon tubes containing pre-equilibrated Strep-Tactin^®^ Superflow resin (IBA Lifesciences) and incubated with gentle rotation at 4 °C for 2 h. The resin was washed with 10 column volumes of wash buffer containing 20 mM HEPES pH 7.5, 200 mM NaCl, 0.1% DDM and eluted with elution buffer containing 20 mM HEPES pH 7.5, 200 mM NaCl, 50 mM D-biotin (Alfa Aesar), 0.05% DDM. The eluted protein was exchanged into a buffer containing 20 mM HEPES pH 7.5, 200 mM NaCl, 0.05% DDM using a PD-10 desalting column (GE), and concentrated down using a 100-kDa concentrator (Pierce) to ~1 mg mL^−1^.

The protein was then incorporated into lipid nanodisc^44^ with a molar ratio 1:300:6 between EmbB:1-palmitoyl-2-oleoyl-sn-glycero-3-phospho-(1′-rac-glycerol) (POPG) (Avanti):membrane scaffold protein 1E3D1 (MSP1E3D1) and incubated for 2 h with gentle agitation at 4 °C. The POPG was prepared by adding the solid extract to deionized water to a final concentration of 20 mM. The mix was placed on ice and then gently sonicated with a tip sonicator (Fisher Scientific) to dissolve the lipids. The lowest power setting was used and sonication was stopped when the mixture turned from cloudy to semi-transparent, after approximately five cycles. No detergent was added to the lipid extract. Reconstitution was initiated by removing detergent with the addition of 150 mg Bio-beads (Bio-Rad) per mL of protein solution for overnight with constant rotation at 4 °C. Bio-beads were removed by passing the protein solution through an Ultrafree centrifugal filter unit (Fisher) at 4000 × *g* in a Centrifuge 5424R (Eppendorf) at 4 °C for 1 min and the nanodisc reconstitution mixture was re-bound to fresh Strep-Tactin^®^ Superflow resin for 2 h at 4 °C in order to remove empty nanodisc. The resin was washed with 10 column volumes of wash buffer consisting of 20 mM HEPES pH 7.5, 200 mM NaCl, followed by three column volumes of elution buffer consisting of 20 mM HEPES pH 7.5, 200 mM NaCl, and 50 mM D-biotin.

The eluent was concentrated using a 100-kDa concentrator to under 500 μL and loaded onto a Superdex 200 Increase 10/300 GL size-exclusion column (GE Healthcare Life Sciences) in gel filtration buffer (20 mM HEPES pH 7.5 and 200 mM NaCl). Throughout the entire process of purification, 15 μL of samples were taken and added to 5 μL of 6X reducing Laemmli SDS sample buffer (Bioland Scientific). The samples were then loaded on a 4–20% Mini-PROTEAN TGX precast protein gel (Bio-Rad) for protein gel electrophoresis in a Tris/Glycine/SDS buffer. The gel was developed using InstantBlue (Sigma) protein stain.

### Negative stain electron microscopy

Purified EmbB in nanodiscs was diluted to 0.005 mg/ml and applied onto copper grids (Ted Pella). These grids were overlaid by a thin (∼1.5 nm) layer of continuous carbon that had been plasma-cleaned (Gatan Solarus) for 30 s using a mixture of H_2_ and O_2_. Thereafter, filter paper (Whatman 4) was used to remove the protein solution. Three microliters of 2% uranyl formate was then added and immediately removed by absorbing with filter paper—this was repeated seven times. The grid was imaged on a Tecnai TF20 microscope (FEI) equipped with a Tietz F416 CCD camera (Tietz) at 1.10 Å per pixel, respectively, using the Leginon software package^45^. Seventy seven images were collected and processed using the Appion software package^46^ to obtain 2D classes with Relion 2.1^47,48^. The micrographs showed good particle dispersion and homogeneity.

### Single-particle Cryo-EM sample vitrification

Purified EmbB was concentrated using a 100-kDa concentrator (Pierce) between 5 and 20 μL of sample at ~8 mg mL^−1^. 1 mM of ethambutol (Sigma) was added before vitrification. 2.5 μL of sample was added to a plasma-cleaned (Gatan Solarus) 0.6/1.0 µm holey gold grid (Quantifoil UltrAuFoil) and blotted using filter paper on one side for 2 s using the Leica GP plunger system before plunging immediately into liquid ethane for vitrification. The plunger was operating at 5 °C with >80% humidity to minimize evaporation and sample degradation.

### Data acquisition

Images were recorded in two sessions. The first session was on a Titan Krios electron microscope (FEI) equipped with a Falcon III direct detector operating at 0.665 Å per pixel in electron counting mode using the Leginon software package^45^. Pixel size was calibrated after obtaining a preliminary map by docking with a crystal structure of a homolog of the soluble part of EmbB (PDB ID: 3PTY [10.2210/pdb3PTY/pdb])^11^. Data collection was performed using a dose of ~78.02 e^−^ Å^−2^ across 80 frames (1080 ms per frame) at a dose rate of ~0.40 e^–^ pix^−1^ s^−1^, using a set defocus range of −0.5 to −2.5 μm. In all, 100-μm objective aperture was used. A total of 2158 micrographs were recorded over 3 days using an image beam shift data collection strategy^49^.

The second session was on a Titan Krios electron microscope (FEI) equipped with a K2 summit direct detector operating at 0.667 Å per pixel in counting mode using the Leginon software package^45^. Pixel size was calibrated in-house using a proteasome test sample. Data collection was performed using a dose of ~77.53 e^−^ Å^−2^ across 80 frames (100 ms per frame) at a dose rate of ~4.3 e^–^ pix^−1^ s^−1^, using a set defocus range of −0.3 to −2.9 μm. In all, 100-μm objective aperture was used. A total of 7833 micrographs were recorded over three days using an image beam shift data collection strategy^49^.

During data collection, movie frames were aligned using MotionCor2^50^ with 5 by 5 patches and B-factor of 100 through the Appion software package^46^. Micrograph CTF estimation was performed using both CTFFind4^51^ and GCTF^52^, and best estimate based on confidence was selected within the Appion software package. The aligned frames and corresponding CTF allowed for monitoring of the collection process in real time.

### Data processing

Data from the two sessions were processed separately and combined toward the end of the processing pipeline. For the first Falcon III dataset, movie frames were aligned using MotionCor2^50^ with 5 by 5 patches and B-factor of 500 for global alignment and 100 for local alignment through the Relion package^47,48^. Micrograph CTF estimates were imported from Appion. Ice thickness measurements were used to filter out micrographs containing ice thicker than 100 nm^53^. Template-free particle picking with Gautomatch (Kai Zhang, unpublished, https://www.mrc-lmb.cam.ac.uk/kzhang/Gautomatch/) using an extremely lenient threshold (to avoid missing any particles) was used to pick particles (extracted 384 box size binned to 256) that were transferred into Relion 2.1 for 2D classification. 2D class averages that were ice or showed no features were discarded, resulting in 162,271 particles. The particle stack was then brought into CryoSPARC^54^ where repeated rounds of two class ab initio and 2D classification were used to clean up the particle stack down to 19,755 particles. The repeated rounds of two class ab initio classification was necessary because of the slight preferred orientation of EmbB—this allowed for the trimming of the dominant views while retaining the less populated ones to give a more directional isotropic reconstruction. GCTF was then used to estimate per-particle CTF and the resulting particle stack was refined to 4 Å in resolution using CryoSPARC non-uniform refinement^55^. Particle polishing was then performed on the particle stack through Relion. The polished particle stack was then put through *cis*TEM^56^ for CTF refinement to obtain better defocus values. The particles with the refined defocus values were then put through another round of CryoSPARC ab initio to further clean up the particle stack and a final non-uniform refinement produced a 3.4 Å map.

For the second K2 summit dataset, movie frames were aligned using MotionCor2 with 5 by 5 patches and B-factor of 500 for global alignment and 100 for local alignment through the Relion package. Micrograph CTF estimates were imported from Appion. Ice thickness measurements were used to filter out micrographs containing ice thicker than 100 nm. Template-free particle picking with Gautomatch using an extremely lenient threshold (to avoid missing any particles) was used to pick particles (extracted 384 box size binned to 256) that were transferred into Relion 2.1 for 2D classification. 2D class averages that were ice or showed no features were discarded, resulting in 700,201 particles. The particle stack was then brought into CryoSPARC where repeated rounds of two class ab initio and 2D classification were used to clean up the particle stack down to 39,702 particles, for the same rationale as stated for the first dataset. GCTF was then used to estimate per-particle CTF^52^. Particle polishing was not done for this dataset. The particle stack was then put through *cis*TEM^56^ for CTF refinement to obtain better defocus values. The particles with the refined defocus values were then put through another round of CryoSPARC ab initio to further clean up the particle stack and a final non-uniform refinement produced a 3.3 Å map.

At this point, both datasets were combined, which was possible because of almost identical pixel size between them (0.9975 Å versus 1.0005 Å). A common pixel size value of 1.00 Å was used for this combined dataset of 57,970 particles. Non-uniform refinement in CryoSPARC produced a 3.3 Å final map, which was locally sharpened with a b-factor of −72.5 Å^2^. Although resolution did not improve after combining, the map features look slightly better in the combined map versus the individual maps from either camera, hence the final map combined both stacks was used.

All conversions between Relion, CryoSPARC, and *cis*TEM were performed using Daniel Asarnow’s pyem script (10.5281/zenodo.3576630).

One millimolar of the drug ethambutol was added before vitrification for this dataset—however, when data was collected without the drug, a 3.7 Å reconstruction was obtained and when compared to the 3.4 Å reconstruction where the drug was added, no differences were observed. Hence the higher resolution map was used for analysis and model building.

### Model building and refinement

Density modification was applied to the map using phenix.resolve_cryo_em^57^. The crystallized structure of the C-terminal soluble domain of EmbC^11^ was docked into EmbB with Chimera^58^ and used as a starting point for model building. Coot^59^ was used for manual model building. After the model was built, it was refined against the cryo-EM map utilizing real space refinement in the Phenix program^60,61^. Restraints for the lipids were generated using phenix.eLBOW and for the metal ions using phenix.ready_set. Thereafter, model adjustment and refinement were performed iteratively in Coot and Phenix, with the statistics being examined using Molprobity^62^ until no further improvements were observed. Residues 1–20, 501–525, and the C-terminal purification tag had poor density and were not built in the model. The final map and model were then validated using (1) EMRinger^63^ to compare a map with a model, (2) CryoSPARC’s blocres implementation^64^ to calculate map local resolution, (3) 3DFSC program suite^65^ to calculate degree of directional resolution anisotropy through the 3DFSC, and (4) SCF program^66^ to calculate the sampling compensation factor (SCF), which quantifies how inhomogeneity in Euler angle distributions contributes to attenuation of the FSC. Map-to-model FSCs were also calculated by first converting the model to a map using Chimera molmap function at Nyquist resolution (2 Å). A mask was made from this map using Relion (after low-pass filtering to 8 Å, extending by 1 pixel and applying a cosine-edge of 3 pixels), and was then applied to the density map. Map-to-model FSC was calculated using EMAN^67^ proc3d between these maps.

### Model analysis

A cavity search using the Solvent Extractor from Voss Volume Voxelator server^68^ was performed using an outer probe radius of 5 Å and inner probe radius of 2 Å. In order to search for other PDB structures with similar fold, a Dali server^25^ search was performed—first globally and then against the different domains of the model. The Dali server was used to generate the structural conservation figures. Coot SSM superpose was used to align structures of other glycosyltransferases against EmbB. ConSurf^69^ was used for generating sequence conservation data for the structure.

### Mass spectrometry

EmbB was buffer exchanged into 0.2 M ammonium acetate at pH 6.8 (Sigma-Aldrich) with either 0.01% C12E8 (Anatrace) or 0.02% DDM (Anatrace) detergent using gel filtration. Native mass spectrometry (MS) analysis of EmbB in C12E8 detergent was performed using a Q-Exactive HF Orbitrap with Ultra High Mass Range modifications (Thermo Fisher Scientific) using previously described methods^70^. The HCD voltage was set to 150 V, and the capillary temperature was increased to 300 °C. Denatured intact LC-MS was performed on EmbB in DDM using a SolariX FTICR mass spectrometer (Bruker). The online LC separation was performed using a BioResolve RP mAB polyphenyl, 450 Å, 2.7 µM, 2.1 × 100 mm column (Waters) with the column temperature at 65 °C. The gradient was adjusted over 38 min from water to acetonitrile, each with 0.1% formic acid. The protein eluted at around 30/70 water/acetonitrile. For both native and denatured mass spectra, data were deconvolved and analyzed using UniDec^71^. Uncertainties were derived from the weighted standard deviation of masses measured at different charge states.

### Screening, imaging, and data analysis of the glycan array

Glycan array analysis was done with *M. smegmatis* EmbB protein solubilized in DDM. Slides were prewetted in buffer A (25 mM Tris-HCl pH 7.8, 0.15 mM NaCl, 2 mM CaCl_2_, and 0.05% Tween 20) for 5 min, rinsed with buffer B (25 mM Tris-HCl pH 7.8, 0.15 mM NaCl, and 2 mM CaCl_2_) three times, and blocked overnight with buffer C (1% BSA in 25 mM Tris-HCl pH 7.8, 0.15 mM NaCl, and 2 mM CaCl_2_) at 4 °C. Aliquots (500 μL) of serial dilutions of protein samples in buffer C were transferred to wells of the slide module immediately after aspiration of the blocking buffer. Wells were sealed with an adhesive seal and incubated for 60 min at 37 °C. Protein was removed by aspiration, and slides were washed 10 times with buffer A and three times with buffer B. Fluorescence was measured directly or after addition of a secondary antibody in buffer C (1:1000 dilution). Slides were incubated with a secondary antibody at room temperature for 40 min before being washed repeatedly with buffer A and deionized water.

Before being scanned, slides were dried by centrifugation. Microarrays were scanned at 5-μm resolution with a GenePix 4000B scanner (Molecular Devices, Sunnyvale, CA). The fluorescent signal was detected at 532 nm for Cy3 or Alexa Fluor 555 and 488 nm for Alexa Fluor 488. The laser power was 100%, and the photomultiplier tube gain was 400. The fluorescent signals were analyzed by quantifying the pixel density (intensity) of each spot using GenePix ProMicroarray Image Analysis Software version 6.1. Fluorescence intensity values for each spot and its background were calculated. The local background signal was automatically subtracted from the signal of each separate spot, and the mean signal intensity of each spot was used for data analysis. Averages of triplicate experiments and standard deviations were calculated using Microsoft Excel.

### Reporting summary

Further information on research design is available in the Nature Research Reporting Summary linked to this article.

## Supplementary information

Supplementary Information

Peer Review File

Reporting Summary

## Data Availability

All raw movie frames, micrographs, the particle stack and relevant metadata files has been deposited into EMPIAR^72^ as EMPIAR-10420. The electron density map has been deposited into EMDB^73^ as EMD-21983. The model has been deposited into PDB^74^ as 6X0O [10.2210/pdb6X0O/pdb]. All other data are available in the paper or the supplementary materials.
