# Supplementary material for: Cryo-EM structure of arabinosyltransferase EmbB from Mycobacterium smegmatis
Source: Nat Commun. 2020 Jul 7;11:3396. doi: 10.1038/s41467-020-17202-8 (PMC7341804; doi:10.1038/s41467-020-17202-8)
Supplement: Supplementary file 1 — Supplementary Information [file 41467_2020_17202_MOESM1_ESM.pdf]

Supplementary Materials

Cryo-EM Structure of Arabinosyltransferase EmbB from *Mycobacterium smegmatis*

Tan *et al.*

Supplementary Figures 1-10

Supplementary Tables 1-2

Supplementary References

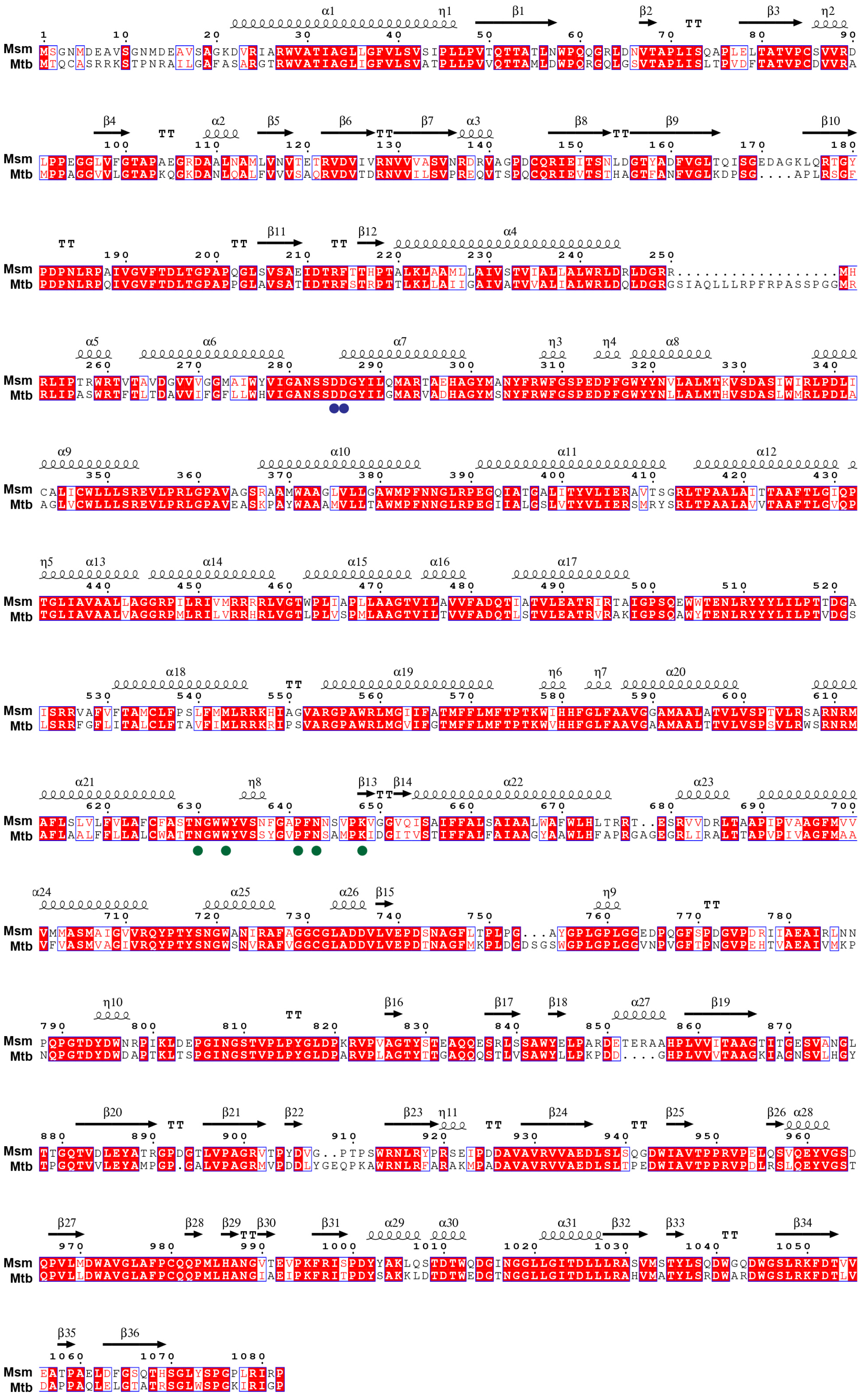

Supplementary Figure 1. Sequence alignment of EmbB from *M. smegmatis* versus *M. tuberculosis.*

The sequence alignment between *M. smegmatis* EmbB (MSMEG_6389) and *M. tuberculosis* EmbB (Rv3795) was generated using ClustalO[1] and displayed using ESPript[2]. Secondary structure annotation based on the EmbB structure is displayed above the sequences. Identical residues are depicted in a red-filled box with white font, whereas residues with more than 70% similarity are marked in a blue bordered box with red font. Blue dots indicate the two catalytic aspartic acid residues, and green dots indicate the residues that when mutated retain enzymatic activity in the enzyme yet reduce incorporation of arabinose.

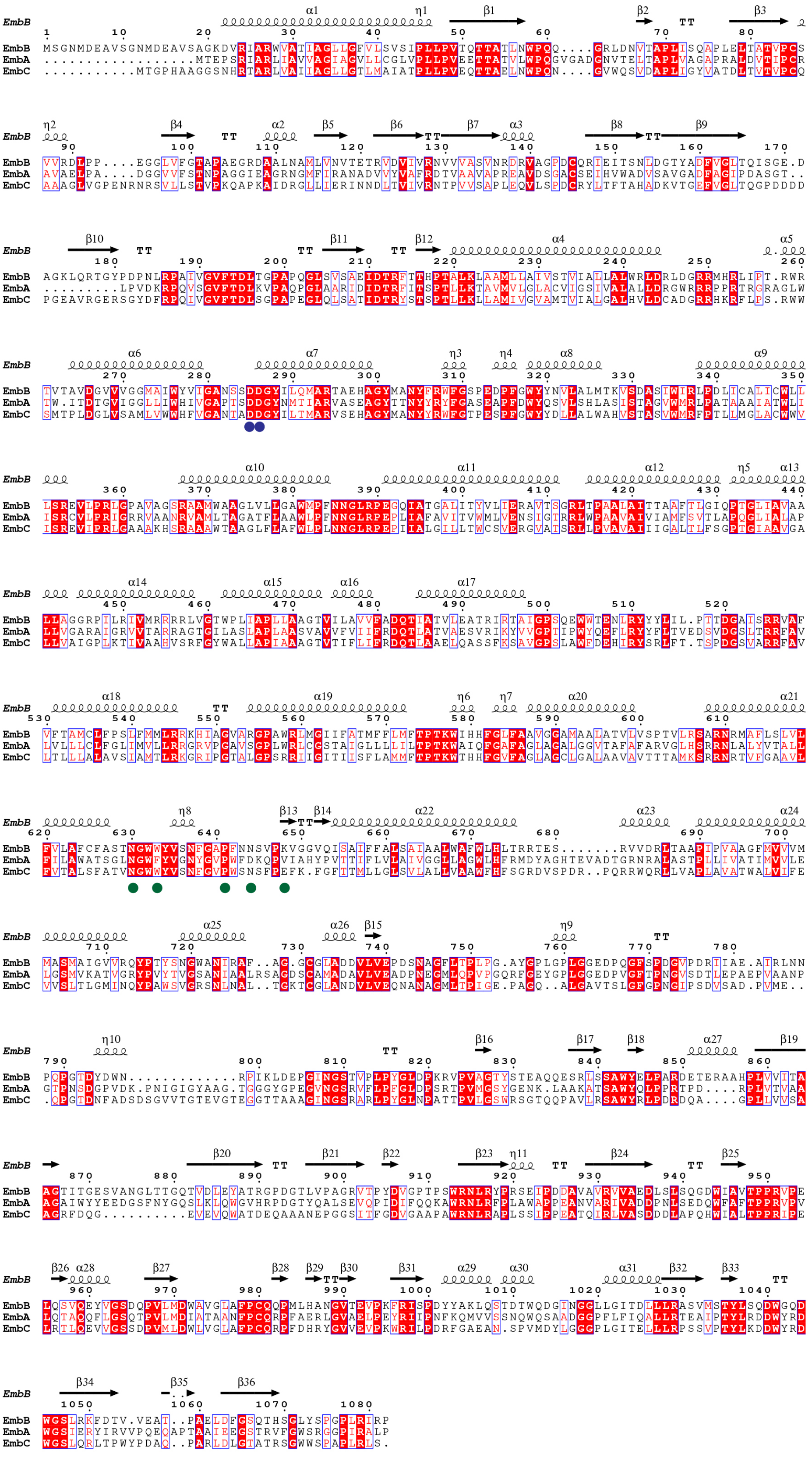

Supplementary Figure 2. Sequence alignment of *M. smegmatis* EmbA, EmbB and EmbC

The sequence alignment between *M. smegmatis* EmbB (MSMEG_6389), EmbA (MSMEG_6388) and EmbC (MSMEG_6387), was generated using ClustalO[1] and displayed using ESPript[2]. Secondary structure annotation from the EmbB structure is displayed above the sequences. Identical residues are marked in a red filled box with white font, while residues above 70% similarity are depicted in a blue bordered box with red font. Blue dots indicate the two catalytic aspartic acid residues, and green dots indicate the residues that when mutated retain enzymatic activity in the enzyme yet reduce incorporation of arabinose.

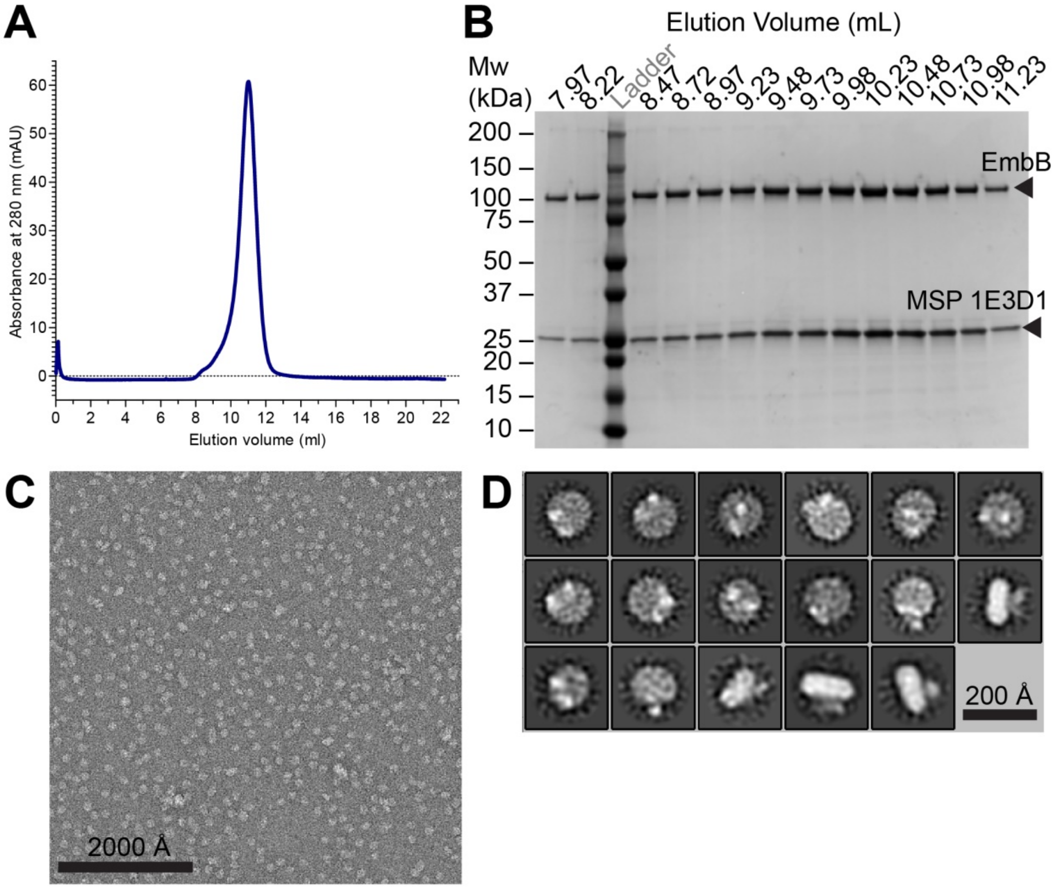

Supplementary Figure 3. Purification and Negative Stain Electron Microscopy of EmbB

(A) Representative size-exclusion chromatography trace of EmbB incorporated into MSP-1E3D1 nanodiscs. Fractions corresponding to the peak were used for cryo-EM analysis. (B) SDS-PAGE of concentrated sample from the peak fractions stained with Coomassie blue. EmbB has a predicted molecular weight of 117 kDa and MSP-1E3D1 of 32 kDa. (C) Representative negative stain micrograph of EmbB. (D) 2D negative stain class averages of EmbB.

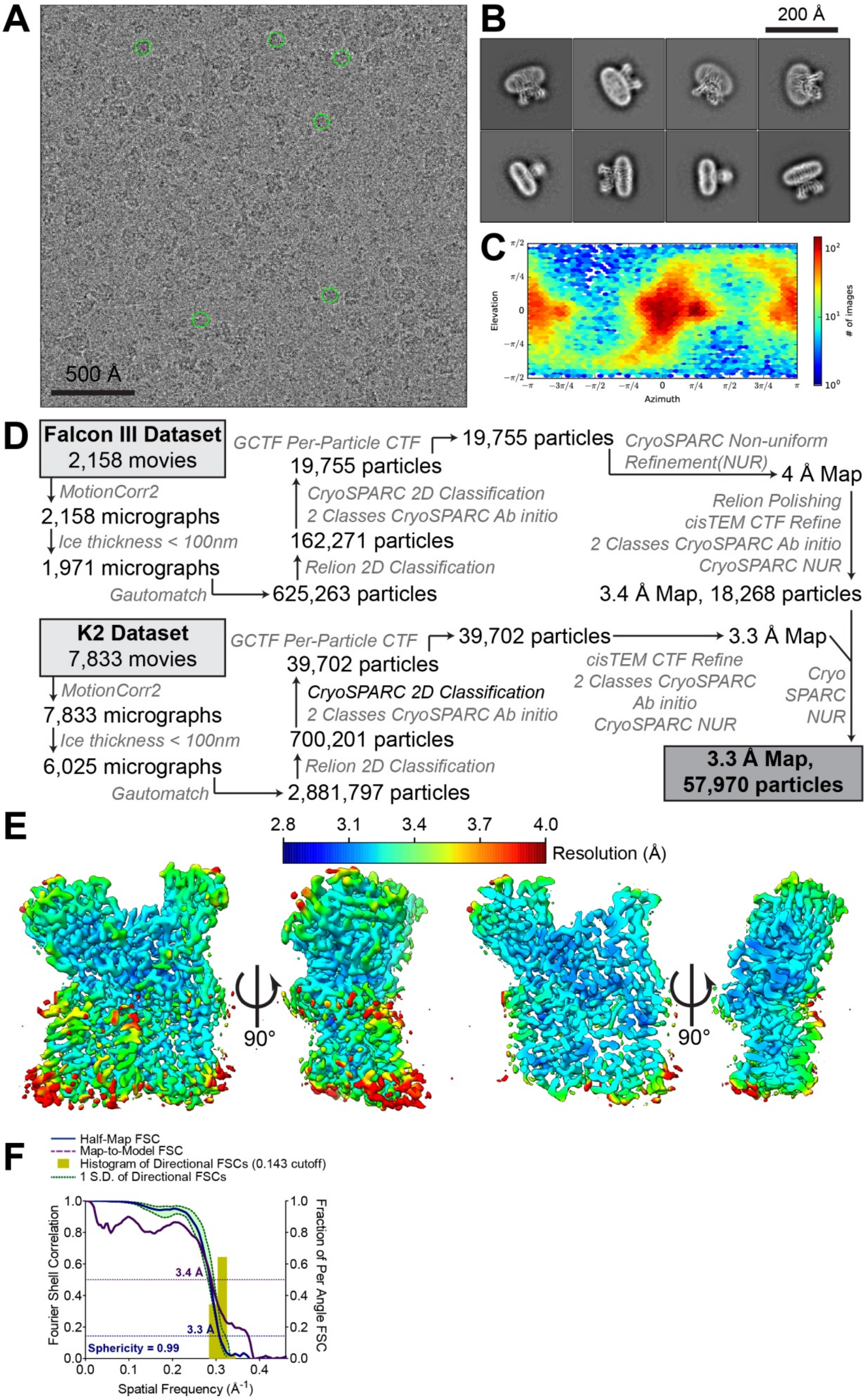
A
500 Å
B
200 Å
C
Elevation
Azimuth
# of images
D
Falcon III Dataset
2,158 movies
MotionCorr2
2,158 micrographs
Ice thickness < 100nm
1,971 micrographs
Gautomatch
625,263 particles
Relion 2D Classification
162,271 particles
CryoSPARC 2D Classification
2 Classes CryoSPARC Ab initio
19,755 particles
GCTF Per-Particle CTF
19,755 particles
CryoSPARC Non-uniform Refinement(NUR)
4 Å Map
Relion Polishing
cisTEM CTF Refine
2 Classes CryoSPARC Ab initio
CryoSPARC NUR
3.4 Å Map, 18,268 particles
K2 Dataset
7,833 movies
MotionCorr2
7,833 micrographs
Ice thickness < 100nm
6,025 micrographs
Gautomatch
2,881,797 particles
Relion 2D Classification
700,201 particles
CryoSPARC 2D Classification
2 Classes CryoSPARC Ab initio
39,702 particles
GCTF Per-Particle CTF
39,702 particles
3.3 Å Map
cisTEM CTF Refine
2 Classes CryoSPARC Ab initio
CryoSPARC NUR
Cryo SPARC NUR
3.3 Å Map, 57,970 particles
E
2.8 3.1 3.4 3.7 4.0
Resolution (Å)
90°
90°
F
Half-Map FSC
Map-to-Model FSC
Histogram of Directional FSCs (0.143 cutoff)
1 S.D. of Directional FSCs
Fourier Shell Correlation
Fraction of Per Angle FSC
3.4 Å
3.3 Å
Sphericity = 0.99
Spatial Frequency (Å-1)

Supplementary Figure 4. Single-particle cryo-EM structural determination of EmbB

(A) Representative micrograph from the first session of imaging using a Falcon III camera. The exposure image had a defocus of –1.9 µm (estimated by CTFFind4) and pixel size of 0.665 Å/pixel. Particles that went into the final reconstructions are circled in green. (B) Representative 2D class averages. (C) Euler angle distribution plot of the final 3D reconstruction. (D) Processing workflow for both cryo-EM datasets. (E) Local resolution display of EmbB reconstructions in orthogonal views. (F) Fourier shell correlation (FSC) curves for EmbB describing the half-map (blue) and map-to-model (purple) resolutions (at 0.143 and 0.5 cut-offs respectively), as well as histogram of directional resolutions sampled evenly over the 3DFSC (yellow). The corresponding sphericity value is also indicated.

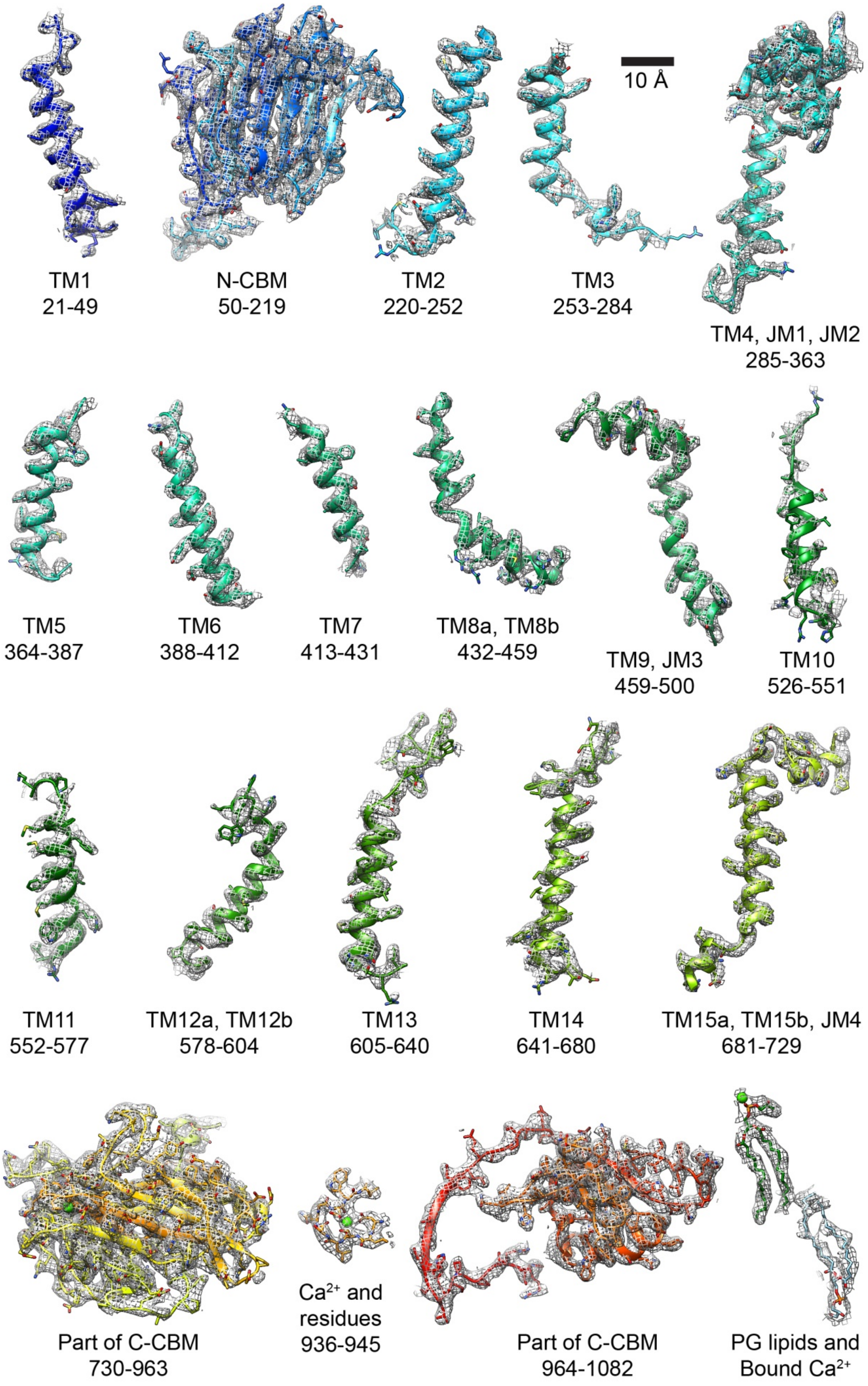
10 Å
TM1
21-49
N-CBM
50-219
TM2
220-252
TM3
253-284
TM4, JM1, JM2
285-363
TM5
364-387
TM6
388-412
TM7
413-431
TM8a, TM8b
432-459
TM9, JM3
459-500
TM10
526-551
TM11
552-577
TM12a, TM12b
578-604
TM13
605-640
TM14
641-680
TM15a, TM15b, JM4
681-729
Part of C-CBM
730-963
Ca^{2+} and
residues
936-945
Part of C-CBM
964-1082
PG lipids and
Bound Ca^{2+}

Supplementary Figure 5. EM Density of EmbB

The atomic model for the structure of EmbB is colored in rainbow and rendered as a cartoon, with the side chains rendered as sticks. The map density is displayed as a mesh. The residues for each segment of the atomic model are indicated below the name of the segment. For all the segments except the PG lipids, the sigma value for display was 0.05, while for the PG lipids, it was 0.02.

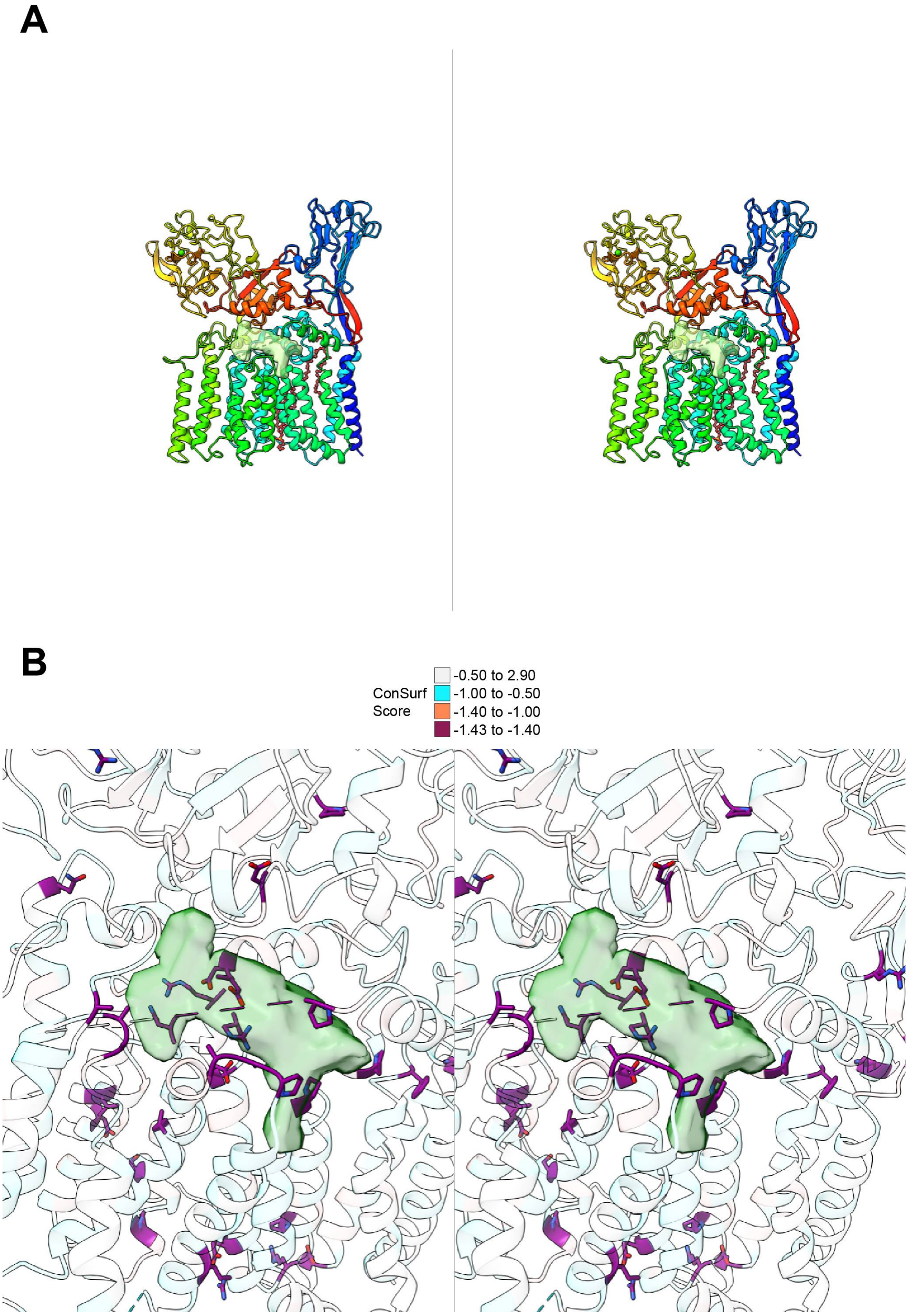
A
B
ConSurf
Score
-0.50 to 2.90
-1.00 to -0.50
-1.40 to -1.00
-1.43 to -1.40

Supplementary Figure 6. Stereoscopic View of EmbB

(A) Walleye representation of EmbB architecture as depicted in Fig. 1c. (B) Walleye representation of EmbB's active site as depicted in Fig. 2b.

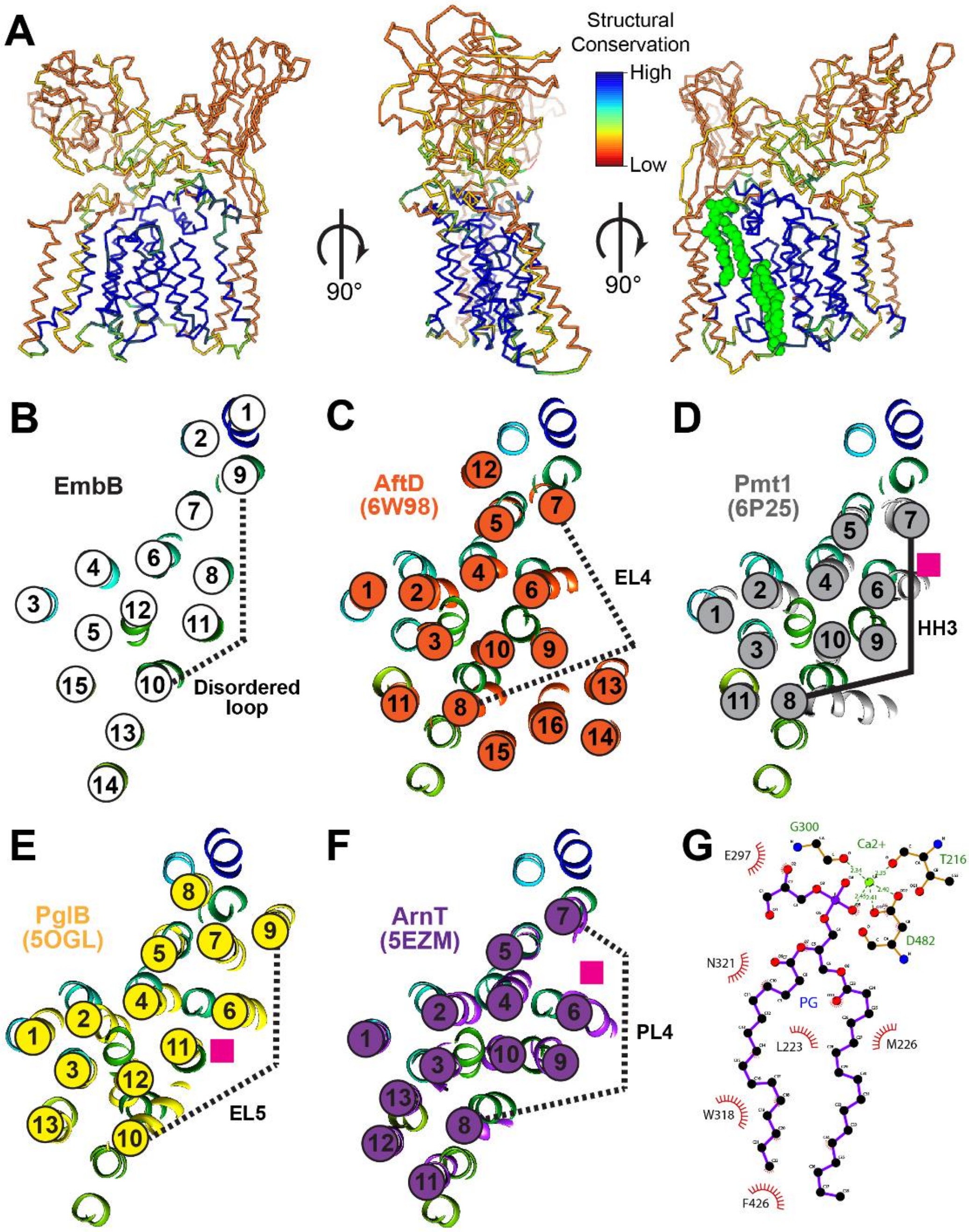

A
Structural Conservation
High
Low
90°
90°
B
EmbB
Disordered loop
C
AftD (6W98)
EL4
D
Pmt1 (6P25)
HH3
E
PglB (5OGL)
EL5
F
ArnT (5EZM)
PL4
G
G300
Ca2+
E297
T216
D482
N321
PG
L223
M226
W318
F426

Supplementary Figure 7. Comparison of TM helices of EmbB with other GT-C Structures

(A) Structural conservation of EmbB (GT53 family) against representative GT-C glycosyltransferases AftD (GT53 family, PDB ID: 6W98), Pmt1-Pmt2 (GT39 family, PDB ID: 6P2R), PglB (GT66 family, PDB ID: 5OGL) and ArnT (GT83 family, PDB ID: 5EZM). The protein is shown in cartoon, and the two PG lipids are shown in the last diagram as green spheres. Structural alignment was performed by the Dali server. Topology of the TM helices of EmbB with reference to the disordered loop is shown in (B). The other glycosyltransferase structures, mycobacterial AftD (C), eukaryotic Pmt1-Pmt2 (D), bacterial PglB (E) and archaeal ArnT (F), were superimposed onto full-length EmbB and a slice through the transmembrane helices are shown as cartoon and colored in orange, grey, yellow and purple, respectively. Locations of the flexible loop that becomes ordered upon substrate binding are indicated by the dotted lines. Pmt1-Pmt2's HH3 is already ordered as a helix without any substrate binding, and hence is shown as a solid line. Locations of lipidic sugar donor substrate are indicated by pink boxes. (G) LigPlot+[3] rendering of the tightly bound PG with Ca^{2+}.

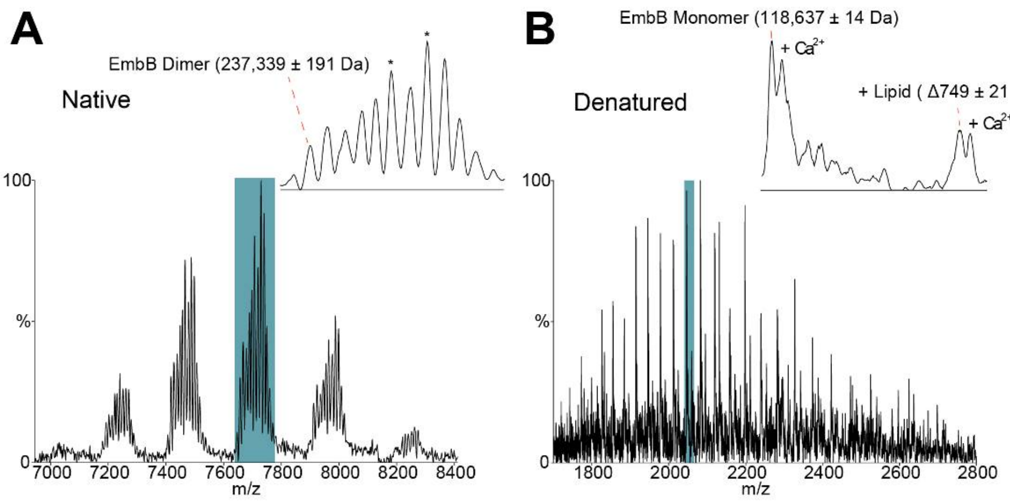

Supplementary Figure 8. Mass Spectrometry of EmbB

Native (A) and denatured (B) mass spectra of EmbB in C12E8 and DDM detergent, respectively. Insets show the regions shaded in *blue*, and key peaks for EmbB monomer, dimer, and adducts are annotated. Peaks in (A) marked with an asterisk likely have two or three bound lipids.

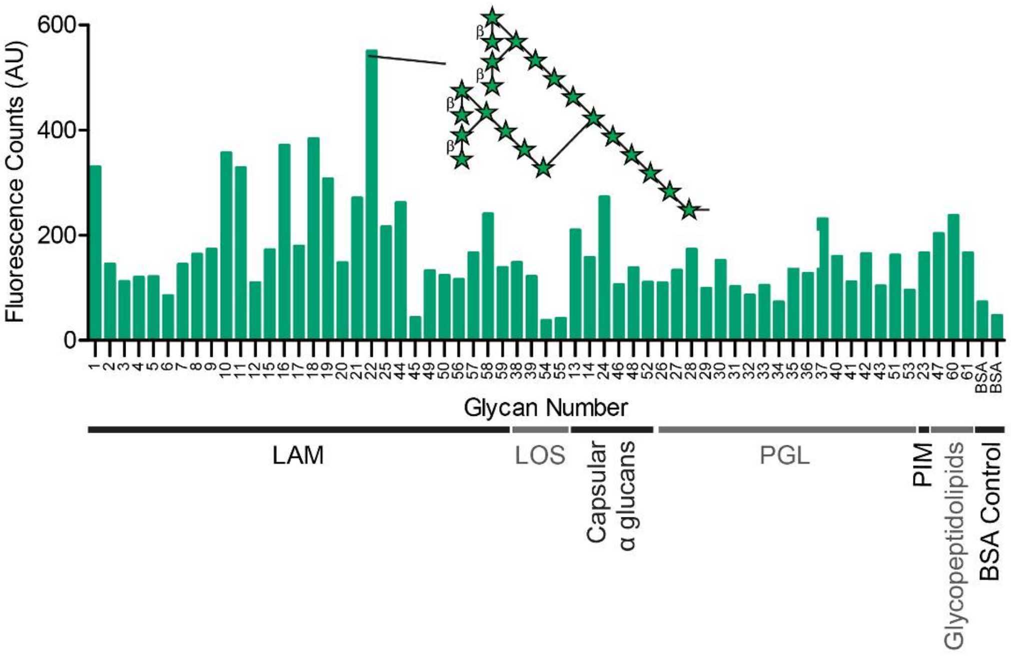

Supplementary Figure 9. Glycan Array Analysis of EmbB

Glycan array analysis of EmbB, with top glycan motif hit indicated, where green star represents arabinofuranose. The classes of glycans used are: lipoarabinomannan (LAM), trehalose mycolates and lipooligosaccharides (LOS), capsular α glucans, phenolic glycolipids (PGL), phosphatidyl-*myo*-inositol mannoside (PIM), glycopeptidolipid and bovine serum albumin (BSA) control. For structures of all of the glycans on the array see [4].

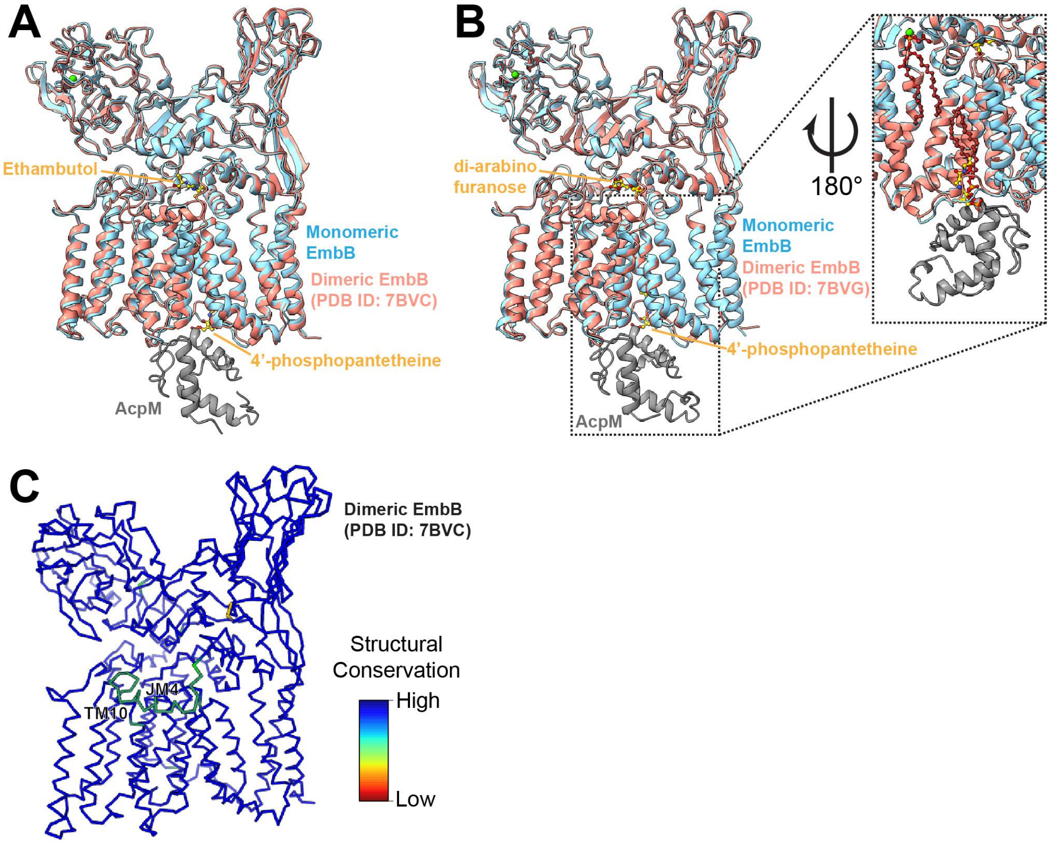

Supplementary Figure 10. Comparison with Structures of Dimeric EmbB

Structures of *M. smegmatis* EmbB solved as a hetero-dimer with EmbA with either ethambutol inhibitor (PDB ID: 7BVC) (A) or di-arabinofuranose ligand (PDB ID: 7BVG) (B) are colored in salmon and superimposed with the monomeric apo-EmbB solved in this paper, colored in pale blue. The bound AcpM is colored in grey, and the ligands colored in gold. Zoom in of the two PG lipids in the monomeric apo-EmbB in brown is shown as an insert in (B). (C) Structural conservation of monomeric EmbB against the two aforementioned dimeric EmbB, mapped onto the structure of the dimeric EmbB (PDB ID: 7BVC). Structural alignment was performed by the Dali server.

Organism Found in	Residue Number in Mtb	Residue in Mtb	Residue Number in Msm	Residue in Msm	Mutated to
***M. tuberculosis* (Mtb)**	297	S	283	S	A[5]
	306	M	292	M	I[5-7]<br>L[5,7]<br>V[5-7]
	319	Y	305	Y	C[8]
	328	D	314	D	G[5]<br>Y[5]
	330	F	316	F	V[5,7]
	334	Y	320	Y	H[5]
	354	D	340	D	A[6]
	406	G	392	G	A[5]<br>C[5,6]<br>D[5,6]<br>S[5,6]
	445	Q	431	Q	R[6]
	497	Q	483	Q	K[5]
	506	T	492	T	R[5,6]
	745	G	729	G	D[5]
	959	D	942	G	A[5]
	1000	M	984	M	R[5]
	1024	D	1008	S	N[5]
	1082	T	1066	S	A[5]
***M. smegmatis* (Msm)**	303	I	289	I	F[9]<br>M[9]
	306	M	292	M	T[9]

Supplementary Table 1. Ethambutol Resistance Mutations Found in EmbB

Residues that are not conserved between EmbBs from *M. tuberculosis* (Mtb) and *M. smegmatis* (Msm) are colored in red.

Residue Number in Mtb EmbC	Residue in Mtb EmbC	Residue Number in Msm EmbC	Residue in Msm EmbC	Residue Number in Msm EmbB	Residue in Msm EmbB	Mutated to
294	D	280	D	286	D	G[10]
300	M	286	M	292	M	L[10]<br>V[10]
394	N	380	N	386	N	D[5]
398	P	384	P	390	P	S[6]
491	Q	477	Q	483	Q	R[6]
502	L	488	F	494	I	P[6]
738	R	719	R	721	W	Q[5]

Supplementary Table 2. Ethambutol Resistance Mutations Found in the *M. tuberculosis* EmbC

Residues that are not conserved among *M. tuberculosis* (Mtb) EmbC and *M. smegmatis* (Msm) EmbB and EmbC are colored in red.

SUPPLEMENTARY REFERENCES

1 Sievers, F. *et al.* Fast, scalable generation of high‐quality protein multiple sequence alignments using Clustal Omega. *Molecular systems biology* **7** (2011).

2 Robert, X. & Gouet, P. Deciphering key features in protein structures with the new ENDscript server. *Nucleic acids research* **42**, W320-W324 (2014).

3 Laskowski, R. A. & Swindells, M. B. (ACS Publications, 2011).

4 Zheng, S. Q. *et al.* MotionCor2: anisotropic correction of beam-induced motion for improved cryo-electron microscopy. *Nat Methods* **14**, 331-332, doi:10.1038/nmeth.4193 (2017).

5 Ramaswamy, S. V. *et al.* Molecular genetic analysis of nucleotide polymorphisms associated with ethambutol resistance in human isolates ofMycobacterium tuberculosis. *Antimicrobial agents and chemotherapy* **44**, 326-336 (2000).

6 Safi, H. *et al.* Evolution of high-level ethambutol-resistant tuberculosis through interacting mutations in decaprenylphosphoryl-β-D-arabinose biosynthetic and utilization pathway genes. *Nature genetics* **45**, 1190 (2013).

7 Sreevatsan, S. *et al.* Ethambutol resistance in Mycobacterium tuberculosis: critical role of embB mutations. *Antimicrobial agents and chemotherapy* **41**, 1677-1681 (1997).

8 Sun, Q. *et al.* Mutations within embCAB are associated with variable level of ethambutol resistance in Mycobacterium tuberculosis isolates from China. *Antimicrobial agents and chemotherapy* **62**, e01279-01217 (2018).

9 Lety, M., Nair, S., Berche, P. & Escuyer, V. A single point mutation in the embB gene is responsible for resistance to ethambutol in Mycobacterium smegmatis. *Antimicrobial agents and chemotherapy* **41**, 2629-2633 (1997).

10 Goude, R., Amin, A., Chatterjee, D. & Parish, T. The arabinosyltransferase EmbC is inhibited by ethambutol in Mycobacterium tuberculosis. *Antimicrobial agents and chemotherapy* **53**, 4138-4146 (2009).
