# Supplementary material for: Cryo-EM structure of arabinosyltransferase EmbB from Mycobacterium smegmatis
Source: Nat Commun. 2020 Jul 7;11:3396. doi: 10.1038/s41467-020-17202-8 (PMC7341804; doi:10.1038/s41467-020-17202-8)
Supplement: Supplementary file 2 — Peer Review File [file 41467_2020_17202_MOESM2_ESM.docx]

Peer Review Information

**Journal:** Nature Communications

**Manuscript title:** Cryo-EM Structure of Arabinosyltransferase EmbB from Mycobacterium smegmatis

**Corresponding author name(s):** Dr Filippo Mancia

**Editorial notes:**

**Transferred manuscripts**

This manuscript has been previously reviewed at another journal that is not operating a transparent peer review scheme. This document only contains reviewer comments and rebuttal letters for versions considered at *Nature Communications*.

**Reviewer comments & decisions:**

**Reviewer comments, first version:**

Reviewer #1 (Remarks to the Author):

The authors have addressed some of the previous comments, but major concerns still stand and in fact the main concern that the M. smegmatis protein is a poor substitute for the M. tuberculosis protein is increased.

In addition to previous concerns, the fact that the Mtb enzyme could not be used because it behaved differently from the Msm enzyme indicates differences in structure. The similarity between the three paralogs of one species is as great as that between the homologs from the different species. These arabinosyl transferases have different functions in cell wall biosynthesis. However, they would not suggest using a paralog for structural inference. Plus, the M.smegmatis homologs are not functionally equivalent to the Mtb homologs.

The fact that they could not determine structures with drug are also concerning, especially in view of their claims that this structure could be used for drug discovery. This is overstated.

**Author rebuttal, first version:**

***Reviewer #1***

*Reviewer #1 (Remarks to the Author):*

*The authors have addressed some of the previous comments, but major concerns still stand and in fact the main concern that the M. smegmatis protein is a poor substitute for the M. tuberculosis protein is increased.*

*In addition to previous concerns, the fact that the Mtb enzyme could not be used because it behaved differently from the Msm enzyme indicates differences in structure. The similarity between the three paralogs of one species is as great as that between the homologs from the different species. These arabinosyl transferases have different functions in cell wall biosynthesis. However, they would not suggest using a paralog for structural inference. Plus, the M.smegmatis homologs are not functionally equivalent to the Mtb homologs.*

We acknowledge this shortcoming of our manuscript and have addressed this issue in its final paragraph.

*The fact that they could not determine structures with drug are also concerning, especially in view of their claims that this structure could be used for drug discovery. This is overstated.*

With regards to a drug bound structure, we have tried different combinations of drug and substrate, but were unable to successfully incorporate them into the structure. Examples are using ethambutol and undecaprenyl phosphoarabinose. It could be that the drug might require the presence of substrate in order to bind, or maybe other co-factors are required. These hypotheses and routes are all being actively explored, however both previous Reviewer 2 and us agree that it does not diminish the structural insights of this paper and that such studies would be a logical future direction.

Additionally, we feel that mapping of the active site and known resistance mutations is highly important and significant, and despite the lack of a ligand, we believe the clustering of mutations is sufficient to allow for insights into the drug binding pocket. Just to cite one of our own examples, in the recent and absent-ligand structure-based report of the malarial transporter for chloroquine (PfCRT), which was published in December 2019 in *Nature*, the drug resistance mutations map unambiguously to a physical location in the structure^1^.

**Literature Cited**

1	Kim, J. *et al.* Structure and drug resistance of the plasmodium falciparum transporter pfcrt. *Nature* **576**, 315-320 (2019).

**Reviewer comments, second version:**

**Author rebuttal, second version:**

**Reviewer comments, third version:**

**Author rebuttal, third version:**
